# Financial well-being and financial literacy in Romania: A survey dataset

**DOI:** 10.1016/j.dib.2022.108413

**Published:** 2022-06-25

**Authors:** Mihai Nițoi, Dorina Clichici, Cristina Zeldea, Miruna Pochea, Cecilia Ciocîrlan

**Affiliations:** a Institute for World Economy, Romanian Academy, Bucharest, Romania; b Department of Finance, Babeş-Bolyai University, Cluj-Napoca, Romania

**Keywords:** Financial well-being, financial behavior, financial knowledge, financial education, Romania

## Abstract

This article presents a dataset aiming to examine the nexus between financial well-being, financial behavior, and financial literacy in Romania. A questionnaire-based survey was conducted on a representative sample of 1,391 respondents, selected through a multistage stratified random procedure. The questionnaire encompasses 34 questions aimed at uncovering individual abilities to manage personal finances, attitudes towards several financial instruments or techniques and financial knowledge. We construct one index for measuring financial well-being and three difficulty-ranked financial literacy indices. The indices are adapted to existing measuring techniques to allow for cross-country comparisons. The dataset connects research on the factors determining financial well-being, socio-economic characteristics or behavioral traits, with the measurement of financial knowledge and skills. The provided data can be used by policy makers in designing a national strategy of financial education, as well as by education providers and practitioners in their teaching process. The academy and researchers could also use this data to conduct inter-country financial analysis or studies at the aggregate level.

## Specifications Table

**Table:** 

Subject	Economics, Econometrics, and Finance
Specific subject area	Behavioral Finance and Economics, Education
Type of data	Raw data in .csv and .dta format and analyzed data in Tables.
How the data were acquired	All data were collected via computer-assisted telephone interviews. All data were converted in .csv and .dta format for formal analysis in Stata or R.
Data format	Raw, Analyzed
Description of data collection	The questionnaire was designed by the project team between August 2^nd^, 2021 and September 30^th^, 2021. It covers topics related to financial behavior, financial attitudes, financial well-being, and financial literacy in Romania. The data were collected between October 1^st^ and November 3^rd^, 2021. A representative sample of 1,391 respondents, aged 16 and over, were interviewed. The sample was selected via a multistage stratified random sample procedure and it is representative for the Romanian population structure with respect to age, gender, and region. The interviews were conducted using the CAPI (Computer-assisted personal interview) method for all respondents. After data collection, a weighting procedure based on the universe description, accounting for gender, residential status, and age was implemented. For the weighting procedure, data from the Romanian National Institute of Statistics were used.
Data source location	Romania
Data accessibility	Data in .csv and .dta format, along with the questionnaire used in the survey, can be obtained from Harvard Dataverse. M. Nițoi, D. Clichici, C. Zeldea, M. Pochea, C. Ciocîrlan, Financial well-being and financial literacy in Romania: A survey dataset, Harvard Dataverse, V2, 2022. https://doi.org/10.7910/DVN/KTLS3P.

## Value of the Data

• The database contains original information, useful for examining several dimensions of financial behavior, financial well-being, and financial literacy in Romania. While there is a vast literature on these topics at international and European level, financial well-being and literacy remain unexplored in Romania. The importance of their investigation is strengthened by the laggard position of Romania in Europe: only 8.26% of individuals have financial literacy knowledge and skills.
• Policy makers, regulators, education practitioners, commercial banks, academia, and researchers can benefit from these data. Specifically, individual data can be used to analyze the determinants of financial well-being, including financial behavior patterns and socio-economic characteristics. For financial education providers, the dataset can be valuable for designing flexible, targeted, and social group-tailored policy instruments.
• Despite its country-level characteristics, the dataset is helpful for cross-country analysis, or it can be used as a benchmark for aggregate empirical studies. For instance, it can be exploited to compare country-specific effects on financial education programs’ effectiveness. The questionnaire can also be used for conducting similar surveys in other emerging countries.
• The questionnaire can be applied in Romania on a yearly basis in order to efficiently evaluate the developments of the financial well-being score, financial literacy levels, and, implicitly, effectiveness of financial literacy policies over time. This would provide an evaluation procedure for the implementation of a financial education national strategy.

## 1 Data Description

This survey dataset provides information on financial behavior, financial well-being, and financial literacy in Romania. The dataset was translated into English and includes variables names and labels that corresponds to the questionnaire. To capture the financial behavior particularities, financial well-being individual score, financial literacy level, and individual sociodemographic characteristics, we included 34 items in the questionnaire. These items are divided in four parts. The following sub-sections briefly discuss these parts.

### 1.1 Sociodemographic variables

The first part includes items labelled from SD1 to SD9 and collects data related to geographic, demographic, and socioeconomic variables. Specifically, items SD1 and SD1a gather data about residential status and residential area size. Moreover, variables labeled NUTS3 and NUTS2 collect data on respondent geographic region at NUTS 2 and NUTS 3 level. Items SD2, SD3, SD4, SD6, and SD8 add data on individual characteristics about gender, age, education, household size, and marital status. Finally, items SD5, SD7, and SD9 refer to individual socioeconomic characteristics, which capture employment, individual net income, and bank account ownership. The sociodemographic information of the sample is listed in Table 1.

**Table 1:** Survey sociodemographic characteristics

Variable	%	N	N weighted
**Gender**			

Male	48	669	672
Female	52	722	719

**Age**			
18-24 years	5	83	76
25-34 years	15	218	211
35-44 years	24	328	337
45-54 years	25	340	350
55-64 years	15	208	203
65+ years	15	214	214

**Education**			

Primary school (4 grades)	1	13	15
Middle school (8 grades)	9	134	125
High school (12 grades)	51	697	707
Bachelor and master education	31	446	433
Post-graduate education	8	101	112

**Employment status**			
Student	2	32	28
Employee	64	898	894
Self-employed	5	63	63
Retired	22	314	309
Unemployed	7	84	97

**Number of household members**			

1	10	139	137
2	37	516	519
3	30	415	409
4 +	23	321	326

**Net income**			

< LEI 1,500 (Approx. < EUR 300)	6	90	88
LEI 1,501 – 3,500 (Approx. EUR 301 - 700)	34	471	475
LEI 3,501 – 5,500 (Approx. EUR 701 – 1,100)	37	521	514
LEI 5,501 – 9 000 (Approx. EUR 1,101 – 1,800)	17	239	238
> LEI 9,001 (Approx. > EUR 1,801)	6	70	76

**Marital status**			

Single	12	179	173
Married	75	1037	1048
Divorced	4	54	52
Consensual union	3	50	47
Widowed	5	71	72

**Have a current bank account**			

Yes	74	1042	1031
No	26	349	360

### 1.2 Financial behavior variables

The second part lists items from I1 to I7 and collects data on financial behavior and attitudes. Item I1 measures financial planning behavior. Items I2, I3, and I4 are related to individual saving behavior. Specifically, I2 collects data on individual preferred financial instruments for savings or investments. Item I3 captures information sources used to make financial decisions. Item I4 collects data on the savings barriers. Item I5 complements previous items and shows individual money behavioral traits in relation to financial instruments and money management. Item I6 measures people trust in financial education providers, while I7 reflects potential concepts of interest for each respondent.

### 1.3 Financial well-being

The third part of the survey includes items from A1_1 to A1_6 and from B1_1 to B1_4. The 10 items are used to measure financial well-being. They are similar to those proposed and used in [1] to measure financial well-being in the United States. Also, in [2] five of these items are used to measure financial well-being in 21 countries. Generally, these items measure if each individual manages to control his/her finances, including the capacity to absorb a financial shock, to fulfill different financial goals and to make financial decisions meant to ensure a happy life. Based upon items’ responses, we derive individual financial well-being score, which is revealed by the variable labelled fwb.

**Fig. 1:**
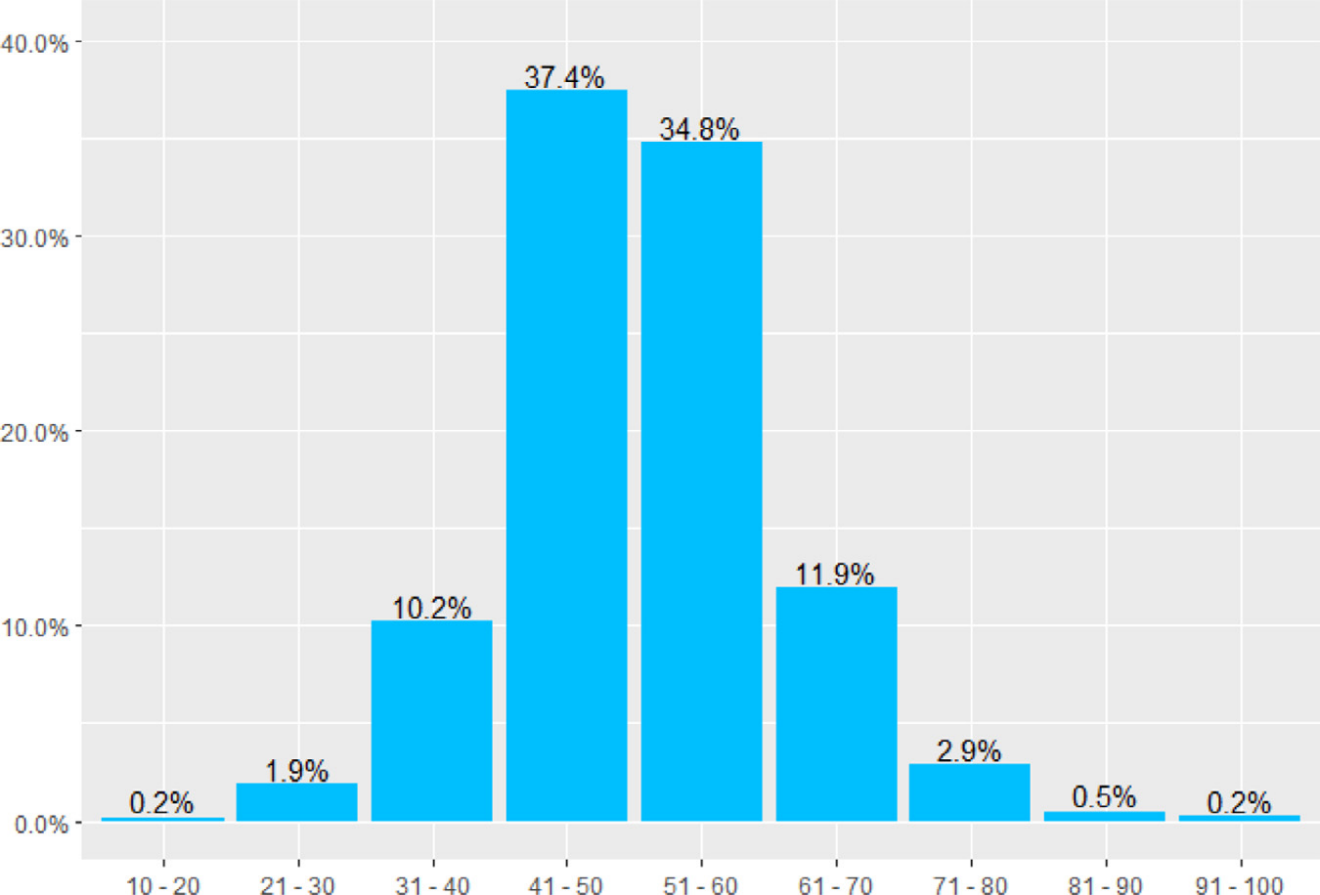
Distribution of the financial well-being values by score ranges

The individual financial well-being index ranges between 16 and 91. Index values ​​of up to 40 indicate that people face a significant level of financial insecurity. Values ​​between 41 and 50 characterize people who do better, but still considerably struggle to make ends meet. Values ​​between 51 and 60 are specific to people that seem to have a medium state of financial stability. Scores higher than 60 are associated with people who have a stable financial situation most of the time. Finally, values ​​higher than 70 characterize people with universal financial security. For Romania, our sample median value is 51. Table 2 and Fig. 1 presents the distribution of financial well-being index by percentiles and score ranges, respectively.

**Table 2:** Distribution of financial well-being values by percentiles

Mean	Percentile 10	Percentile 25	Percentile 50	Percentile 75	Percentile 90
51	39	45	51	57	63

### 1.4 Financial literacy

The final part of the survey comprises eight financial literacy items, labelled from C1 to C8. These items collect data on numeracy skills, basic and advanced financial literacy concepts. The first item measures individual numeracy skills and gathers data on the respondents’ ability to work with simple subtractions and probabilities. The following items are taken and slightly adapted from [3]. Their financial literacy questions became the international standard for measuring individual financial literacy. Items from C2 to C5 are meant to collect data on each respondent knowledge on basic financial concepts. Specifically, they assess individual literacy on interest rate, mortgage loan, money time-value and monetary illusion. The last three questions (items C6, C7, and C8) reflect each respondent knowledge on advanced financial literacy concepts: risk diversification, performance and volatility of financial instruments. We underline that our financial literacy batteries of questions also include the ‘Big Three’ questions (items C2, C5, and C6) proposed in [4], which are used to capture financial literacy level in numerous countries and allow for cross-country comparisons.

Based on individual responses, we report three aggregate financial literacy indices. The first index measures the percentage of individuals who responded correctly to the ‘Big Three’ financial literacy questions (items C2, C5, and C6). The second one reveals the percentage of people who answered correctly to the three advanced financial literacy questions (items C6, C7, and C8). The third shows the percentage of individuals who gave a correct response to all eight financial literacy questions. Table 3 and Table 4 report the three financial literacy indices by percentiles and by number of correct answers.

**Table 3:** The score values of the financial literacy by percentiles

	Mean	Percentile 10	Percentile 25	Percentile 50	Percentile 75	Percentile 90
The ‘Big Three’ questions	1	0	1	1	2	2
The three advanced questions	1	0	0	1	2	2
All eight questions	3	1	2	3	4	5

**Table 4:** Distribution of financial literacy answers

	The ‘Big Three’ questions	The three advanced questions	All the questions
No correct answer	24,7%	37,7%	9,0%
One correct answer	39,3%	31,3%	11,4%
Two correct answers	27,8%	24,8%	18,8%
Three correct answers	8,3%	6,2%	21,0%
Four correct answers			16,9%
Five correct answers			12,9%
Six correct answers			7,1%
Seven correct answers			2,6%
Eight correct answers			0,3%

The findings show that only 8.27% of Romanians answered all ‘Big Three’ financial literacy questions correctly. The result allows for cross-country comparisons, revealing significant differences compared to advanced economies - Germany (53.20%), Switzerland (50.10%), the Netherlands (44.80%), Austria (33.30%), the U.S. (30.20%), France (30.90%), Japan (27%), or Italy (24.90%). Also, the level of financial literacy in Romania is lower, even when compared to that of developing economies such as Albania (9,68%), North Macedonia (10,70%), Serbia (17.11%), Bulgaria (17.94%), Hungary (25.69%), Croatia (27.66%), Poland (29.91%), or Czechia (42.33%). Values for advanced and emerging countries were taken from [5,6].

## 2 Experimental Design, Materials and Methods

The data were acquired by conducting a survey in Romania. The survey questionnaire was prepared between August 2^nd^, 2021, and September 30^th^, 2021 and included 34 items, which collected data on sociodemographic variables, financial behavior, financial well-being, and financial literacy. The data were gathered between October 1^st^, 2021 and November 3^rd^, 2021. A representative sample of 1,391 respondents, aged 16 and over, were interviewed. The sample was selected via a multistage stratified random sample procedure. The sample is representative of the Romanian population structure with respect to age, gender and region. The interviews were held using the CAPI (Computer-assisted personal interview) method for all respondents. After data collection, a weighting procedure based on the universe description, accounting for gender, residential status, and age was implemented. For the weighting procedure, data from the Romanian National Institute of Statistics were used.

The final dataset comprises a number of 73 variables. Most of them were collected based on the survey data. However, some variables were derived from individual responses, e.g., NUTS2, NUTS3, age and age2. Furthermore, the financial well-being score was computed by applying the Item Response Theory to the 10 items related to financial well-being. The calculations were performed in Stata 17. We reordered and labelled variables according to the methodology proposed in [7]. Moreover, to compute individual financial well-being, we created two additional variables – self and age16_61. The former states that the questionnaire was administered by someone else, while the latter reveals the age interval, 16-61 or 62+, for each respondent.

In order to provide a comprehensive picture on financial literacy in Romania, we compute three indices. The first one includes the ‘Big Three’ questions, representing the international standard in measuring financial literacy (items C2, C5, and C6). The second one reveals individual knowledge on advanced financial literacy (items C6, C7, and C8). The last one gathers all eight financial literacy items. For deriving financial literacy indices, each correct answer received a value of one. Afterwards, the results were aggregated for each person and for each financial literacy index. Specifically, for the first and second indices, the financial literacy score will vary in the range of [0, 3], while for the third index, in the range of [0, 8]. The minimum interval value indicates that the respondent did not answer any questions correctly. The maximum value designates the correct answers to all the questions. All these computations were run in Stata 17.

## Ethics Statements

Participation in the survey has been voluntary. Data are fully anonymized. All participants were fully informed and provided their consent. Ethics approval from the institutional committee is not required.

## Declaration of Competing Interest

The authors declare that they have no known competing financial interests or personal relationships that could have appeared to influence the work reported in this paper.

## Data Availability

Financial well-being and financial literacy in Romania: A survey dataset (Original data) (Dataverse).
